# Increased risk of affective disorders in type 2 diabetes is minimized by sulfonylurea and metformin combination: a population-based cohort study

**DOI:** 10.1186/1741-7015-10-150

**Published:** 2012-11-29

**Authors:** Mark L Wahlqvist, Meei-Shyuan Lee, Shao-Yuan Chuang, Chih-Cheng Hsu, Hsin-Ni Tsai, Shu-Han Yu, Hsing-Yi Chang

**Affiliations:** 1 Division of Preventive Medicine and Health Services Research, Institute of Population Health Sciences, National Health Research Institutes, 35 Keyan Road, Zhunan Town, Miaoli, Taiwan 35053, ROC; 2 School of Public Health, National Defense Medical Center, 161 Minchuan East Road, Sec 6, Taipei, Taiwan 114, ROC; 3 Monash Asia Institute, Monash University, 900 Dandenong Road, Caulfield East, Victoria 3145, Australia; 4 Department of Epidemiology and Preventive Medicine, Monash University, 99 Commercial Road, Melbourne, Victoria 3004 Australia; 5 Department of Health Services Administration, China Medical University and Hospital, 91 Hsueh-Shih Road, Taichung, Taiwan 40402, ROC; 6 Department of Psychiatry, Chung Shan Medical University Hospital, 110, Sec 1, Jianguo North Road, Taichung, Taiwan 40201, ROC; 7 Department of Psychiatry, Chung Shan Medical University, No. 110, Sec 1, Chien Kuo N. Road, Taichung, Taiwan 40201, ROC

**Keywords:** Neurodegenerative disease, Taiwan, Mood, Depression, Metformin

## Abstract

**Background:**

To confirm whether type 2 diabetes (T2DM) is an affective disorder (AD) precursor, and to establish possible effects of oral anti-hyperglycemic agents (OAAs).

**Methods:**

A representative cohort of 800,000 subjects was obtained from the Taiwanese National Health Insurance database on 1 January 2000. Those with consistent data (n = 762,753) were followed up between 1 January 1996 and 31 December 2007. Over this period, we assessed the presence (n = 62,988) or absence (n = 699,795) of T2DM, and whether any OAA was used (n = 40,232) or not (n = 22,756). To compare the risk of AD by diabetic status, those with T2DM were matched for birth date and gender with those without T2DM. To assess the effect of OAAs, we considered those 50 years and over. Matched AD-free patients with T2DM on OAAs were compared with those without OAAs, for age, gender, locality, health service, Charlson Comorbidity Index. and diabetes diagnosis date to avoid immortal time bias. AD incidence densities, hazard ratios (HR) and 95% confidence intervals (CIs) were calculated.

**Results:**

Compared with diabetes-free subjects, the HR (95% CI) for AD was 2.62 (2.31 to 2.98) for patients with T2DM who were not on OAAs, and 1.08 (0.99 to 1.18) for those who were on OAAs. The AD incidence density decreased from 91.1 to 39.4 per 10,000 person-years for patients on the combination of metformin and sulfonylurea. The HR (95% CI) for AD was 0.92 (0.59 to 1.45) for those on metformin alone, 1.08 (0.84 to 1.38) for those on sulfonylurea alone, and 0.40 (0.32 to 0.50) for the combined treatment, and the decrease was not related to sequence or insulin usage. Similar patterns were seen for incident AD exclusion for up to 3 years, although more so for bipolar than unipolar.

**Conclusions:**

The incident AD risk is increased by 2.6-fold in T2DM, and the combination of sulfonylurea and metformin minimizes this risk.

## Background

It has been recognized that mood and behavioral disorders may contribute to a co-existent clinical cluster with diabetes [1,2]. Depression and diabetes occur together to a greater extent than would be expected based on their respective separate prevalences [3-9]. There has been uncertainty about the nature of what has been regarded as a bidirectional pathogenesis of the two conditions [10], although a Canadian study failed to find evidence for diabetes being an antecedent for depression [11]. However, studies of European and North American populations do indicate that diabetes is a precursor of depression [12-14]. A meta-analysis estimated that depression is a greater precursor of diabetes than the reverse [15] and, in a Spanish cohort, the incident diabetes rate attributable to depression was 7% [16]. To date, most of these studies have involved Caucasian populations, and there is little information about the issue in those ethnicities that are experiencing rapid and major increases in diabetes prevalence, such as northeast Asians. Over the period 1993 to 2008, the prevalence of diabetes in Taiwan had doubled for men, with an increase in the age-standardized rate from 4.6% to 9.3%, although for women, it decreased from 7.9% to 6.4% [17]. The prevalence of major depression in Taiwan was relatively low at 1.5%, compared with other populations; for instance it was 19.0% in Beirut [18]. However, by 2001, prevalence of major depression in older populations in Taiwan was 5.9% [19], comparable with international figures. Huang's meta-analysis showed that depression was 1.53 times more prevalent among older people with chronic disease [20]. Given the increasing diabetes prevalence, corresponding changes in depression or affective disorder (AD) rates might be expected if there is causality.

The question of anti-hyperglycemic management in the prevention of depression or AD is likely to become an increasingly relevant public-health and clinical matter. There is increasing evidence that the therapeutic scope of the biguanide drug metformin extends beyond its current indications for hyperglycemia to the prevention ofpreviously poorly appreciated outcomes of diabetes. These include certain cancers [21,22] and neurodegenerative disease, notably dementia [23], and Parkinson's disease [24]. There may be a shared pathogenesis between diabetes and dementia related to oxidative stress and mitochondrial dysfunction, together with bioenergetics and abnormal protein folding. Metformin may decrease any or all of these associations. In addition, metformin has been shown to reduce tau hyperphosphorylation, which plays a role in the formation of the neurofibrillary tangles in neurodegeneration [25]. It is conceivable that this might be relevant in neuropsychiatric disorders as well, because tauopathy is seen when depression is co-morbid with Alzheimer's disease [26]. In further support of this concept, the mood-stabilizer lithium also reduces neurofibrillary tangle development [27]. The extent to which these mechanisms are relevant to the development of depression or AD is unknown. Clarification on insulin resistance as a risk setting for depression or AD, and the extent to which this might be avoided, is now of considerable importance as the incidence of glycemic disorders increases globally. Moreover, diabetes and pre-diabetes may each be underlying contributors to depression or AD, because the glycemic disorder progresses with the potentially reinforcing and adverse metabolic effects of depression itself.

The lower directional effect of diabetes on depression compared with the reverse may be due to past non-recognition of risk reduction for depression associated with diabetes therapy [10,12-15]. What may be of related consequence is the potential synergy of depression and diabetes for conventionally characterized diabetes complications such as retinopathy [28]. Thus, it is possible that some studies have not only under-recognized the effect of type 2 diabetes (T2DM) on depression because they have not distinguished between patients treated or not with oral anti-hyperglycemic agents (OAAs), but have also not recognized the mood-modifying potential of anti-hyperglycemic agents.

In this cohort study, we followed up a representative sample of the Taiwanese population for 12 years to determine the effect on risk for AD of T2DM, with and without OAA therapy, using a matching protocol to mimic a clinical trial [24,29,30].

## Methods

### Ethics approval

This study was approved by the institutional review board of the National Health Research Institutes of Taiwan.

### Data sources and study subjects

Taiwan began a national health insurance (NHI) program in 1995. By 2007, 98.4% of Taiwan's 22.96 million population was enrolled in the program. Subject to a double scrambling protocol, the NHI Research Database is based on the registration files and original claims data for reimbursement. The Longitudinal Health Insurance Database 2000 contains all the original claims data of 800,000 beneficiaries, randomly sampled from the year 2000. We studied this database with antecedent data from 1 January 1996.

We conducted two studies with different objectives: the first to determine the effect of diabetes on AD incidence, and the second to ascertain the effect of sulfonylureas or metformin, or a combination of both, on AD.

### Study 1

**Figure 1:**
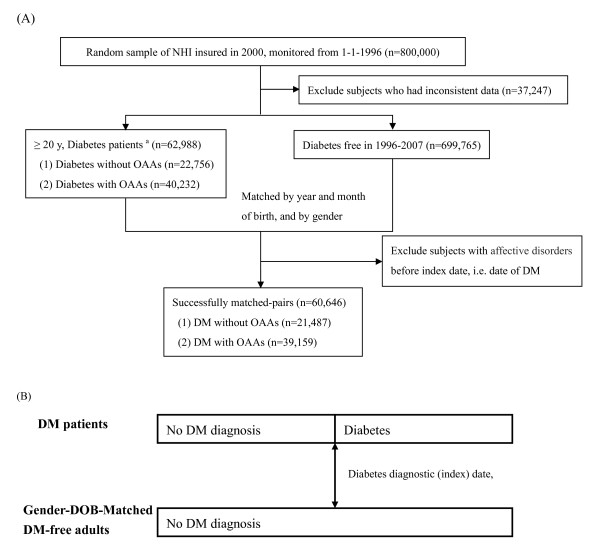
**Patient selection and index date assignment for Study 1**. **(A) **Flow chart of selection of patients with diabetes for comparison of affective disorder (AD) risk with subjects who were free of both diabetes and AD. Subjects aged 20 years or over with a diagnosis of type 2 diabetes mellitus (T2DM) recorded more than once within 1 year or who had used any oral anti-hyperglycemic agent (OAA) for at least 3 months during the period 1996 to 2007 regardless of insulin usage, were evaluated. **(B) **Follow-up of AD onset; patients with diabetes mellitus (DM), who did or did not use oral anti-diabetic agents (OAA). were matched with diabetes-free counterparts, who had the same index dates as the DM diagnostic date.

To determine the effect of diabetes on AD incidence, we first formed a cohort of 762,753 participants with consistent data, who were registered as NHI beneficiaries on 1 January 2000 (Figure 1). They were followed up during the period 1 January 1996 to 31 December 2007. Assessing subjects aged 20 years or above, diabetes was found to be present in 62,988, and absent in the remaining 699,765. Of those with T2DM, 40,232 used any OAA and 22,756 used none. People with T2DM were matched for birth date and gender with those who were diabetes-free, in order to compare AD incidence. Any individual in a matched pair who had previous AD was excluded (Figure 1A). There were 60,646 pairs successfully matched, of which 39,159 pairs were on OAAs and 21,487 pairs were not.

Subjects with T2DM were defined as those who had at least two records of diabetes (ICD-9, CM-250 or A181; the latter was a diabetes code for ambulatory visits used in Taiwan before 2000) within any year during the period 1996 to 2007, or had used any OAA for more than 3 months. In the same matched pair, the subject with T2DM and the T2DM-free subject had the same diabetes diagnostic date (index date) for follow-up (Figure 1B).

The subjects with diabetes were classified into those using or not using OAAs during the follow-up period. We compared the incidence of AD between three groups (T2DM-free, T2DM on OAA, and T2DM without OAA). An AD incident case was defined as one with at least two records of the diagnosis of an AD (A212, or ICD-9 CM coding 296.0 to 296.9, which covers major or unipolar depression (296.2 and 296.3) and bipolar disorders (all other 296.0 to 296.9, but not dysthymic disorders) within any year during the period 1996 to 2007, but after the diagnosis of diabetes. There were 19,009 AD cases in all: 12.073 unipolar and 6.936 bipolar.

### Study 2

**Figure 2:**
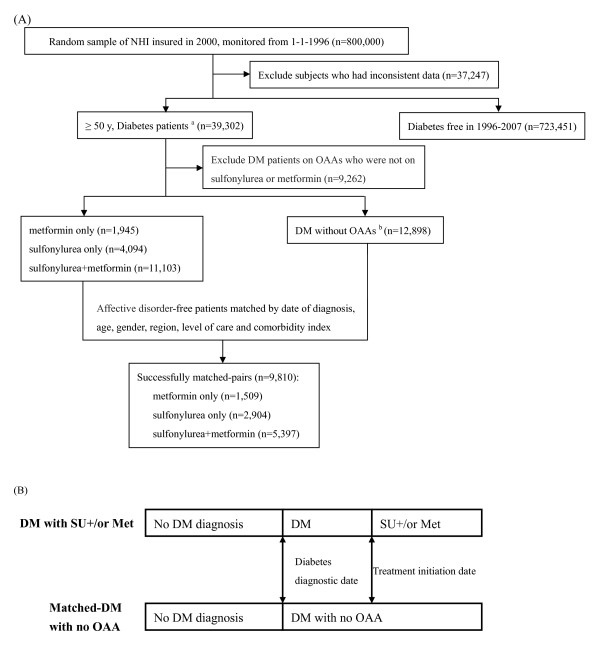
**Patient selection and index date assignment for Study 2**. **(A) **Flow chart of selection of patients with type 2 diabetes who did not have affective disorder (AD) for assessment of AD incidence. Subjects aged 50 years or over with a diagnosis of type 2 diabetes recorded more than once within 1 year or who had used any oral anti-hyperglycemic agent (OAA) for at least 3 months during the period 1996 to 2007, regardless of insulin usage. **(B) **Patients with no OAA exposure were eligible to serve in the matching process for the various OAA groups. Subjects with diabetes on sulfonylureas (SU) and/or metformin (Met) and their matched non-OAA treated counterparts had the same treatment initiation dates (that is, index dates) for follow-up of AD.

We further investigated the preventive effect of the OAAs metformin and/or sulfonylureas for future AD in subjects with T2DM. Metformin or sulfonylureas are the most commonly used OAAs for T2DM treatment in Taiwan. To avoid immortal time bias [24,29,30], we matched patients with T2DM without OAAs and those on metformin or sulfonylureas by age, gender, region, level of care, and Charlson Comorbidity Index (CCI) [31]. In the same matched pair, the subjects with T2DM who were on metformin or sulfonylureas and those who were not on any OAA had the same date (treatment initiation date) for follow-up (Figure 2). Where necessary, a patient in the comparison group was used more than once.

We further classified subjects with T2DM on metformin or sulfonylureas into three groups: metformin only (1,509 pairs), sulfonylureas only (2,904 pairs), and a combination of these two drugs (5,397 pairs). The AD incidences of the three groups were compared with the subjects with T2DM who were not on OAAs (referent group). An insulin user was defined as any subject who had used it for more than 3 months; those who used insulin briefly during hospitalization were not counted as users.

### Matching factors

Subjects' baseline status and comorbidity were treated as matching factors. Comorbidity was measured by the CCI [31] using the NHI diagnoses recorded in the year before the diagnosis of diabetes. For CCI score calculation, we did not include the diagnoses of T2DM because this disease was considered separately in its own right. Other factors included age (in groups aged 50 to 54, 55 to 59 60 to 64, 65 to 69, and ≥70 years), gender, locality, and health service.

### Statistical analysis

The differences between groups were evaluated by one-way ANOVA (CCI score) or χ^2 ^test (demographics). For the effect of diabetes on future AD, the diabetes diagnostic date was used as the starting point to calculate survival time (Figure 1B). For the preventive effect of metformin or sulfonylureas on future depression in subjects with T2DM, the treatment initiation date was used as the starting point to calculate survival time (Figure 2B). The end of follow-up was the onset date of AD or the date of withdrawal from the NHI program or the end of 2007, whichever occurred first. AD incidence density was defined as the number of incident AD events divided by 10,000 person-years at risk.

We used Cox proportional-hazards models to evaluate associations between diabetes, OAAs, and AD. In addition to matching, adjustments were made for monthly income (0, 1 to 15,000, 15,000 to 21000, and >21000 New Taiwan Dollars (NTD)) and use of insulin (yes or no). The statistics software SAS (version 9.1, SAS Institute, Cary, NC, USA) was used for data management and modeling. These models were evaluated using the progressive annual exclusion of incident AD for 3 years after the diagnosis of diabetes. A two-sided *P*-value of < 0.05 was considered significant.

## Results

**Figure 3:**
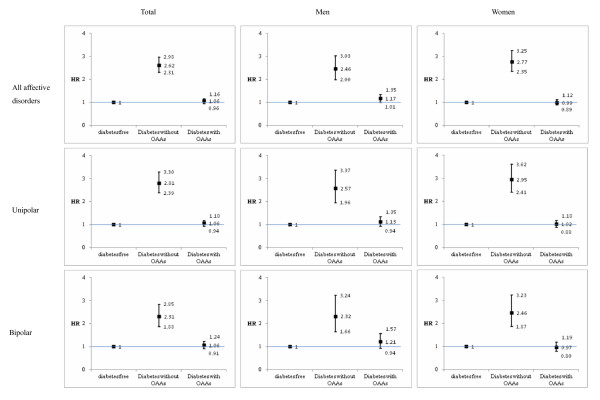
**Relative risks for incident affective disorders by diabetic status and treatment**. Hazard ratios (HR) for incident affective disorders (ADs) by diabetic status without or with oral anti-diabetic agents (OAAs) regardless of insulin usage (using diabetes-free subjects as the comparison group). We matched AD-free subjects with diabetes aged 20 years or over without or with medication with those who were diabetes-free, by year and month of birth and gender. Adjustments were made for monthly income (0, 1 to 15,000, 15,000 to 21000, >21000 New Taiwan Dollars (NTD)).

The hazard ratio (HR) and 95% confidence interval (CI) for AD in those with T2DM and not on OAAs were compared with those who were diabetes-free, and matched for age and gender (Figure 3). The increase in risk was 2.62-fold (95% CI 2.31 to 2.98). For subjects with T2DM who were on any OAA, compared with those who were diabetes-free, the HR of 1.06 (0.96 to 1.16) was not significant, but it represented a normalization of that for T2DM without OAA therapy. The analogous findings by gender and whether AD was unipolar or bipolar are also shown. These indicate that the effects of diabetes and of OAA are similar for men and women and are irrespective of AD polarity.

**Figure 4:**
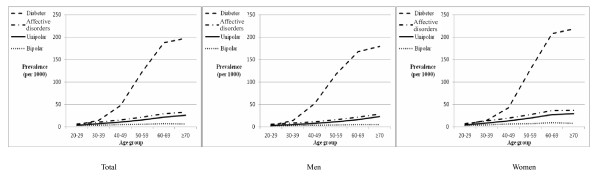
**Prevalence (per 1,000) of diabetes and affective disorders in the year 2000**. Prevalence was stratified by age group.

We calculated the prevalences of T2DM and AD for the year 2000, and plotted them by age (Figure 4). Both prevalences were seen to increase with age, although that for diabetes is more striking than for AD. The steepest increase for diabetes prevalence was between the ages of 40 and 60 years. This informed our choice of 50 years as the age for study 2, as we considered the effects of OAA on the incidence of AD may be more related to diabetes in these subjects than in younger subjects. When each of unipolar and bipolar AD prevalences were considered, those for unipolar AD were found to increase with age, but there was little increase for bipolar AD.

The demographics were stratified according to each metformin or sulfonylurea category, and matched with those with T2DM who had had no OAA (Table 1). There were 9,810 patients with T2DM who were on an OAA and who could be matched with those without an OAA for assessment of AD incidence density and risk of AD. Neither sulfonylureas nor metformin alone were associated with a significant change in AD incidence. For the combination of metformin and sulfonylurea, the incidence density decreased from 91.1 to 39.4 per 10,000 person-years when men and women were considered together, and the findings were similar for both genders (Table 2).

**Table 1:** Demographics for affective disorder (AD)-free patients with diabetes (DM) using oral anti-hyperglycemic agents (OAAs) and their matched counterparts without OAA

	Study 1		Study 2		
	
Descriptor	DM without OAAs (n = 21,487)	DM with OAAs (n = 39,159)	Met only^b ^(n = 1509)	SUs only^c ^(n = 2904)	SUs + Met^d ^(n = 5397)
Age, mean ± SD years					
For matched pair	56.0 ± 15.2	57.5 ± 12.3	67.7 ± 9.1	67.7 ± 9.1	65.7 ± 8.9
At DM diagnosis,	56.0 ± 15.2	57.5 ± 12.3	67.5 ± 8.7	67.5 ± 8.7	65.3 ± 8.2
Age groups, years					
<50	35.8	28.6			
50 to 54	11.9	13.8	6.0	6.8	10.3
55 to 59	11.2	13.9	16.7	16.6	20.2
60 to 64	10.1	13.9	19.3	17.7	20.4
65 to 69	10.4	12.9	18.8	19.4	19.0
≥70	20.6	16.3	39.2	39.6	30.1
Female gender	52.3	48.3	54.5	50.8	52.5
Locality					
North	46.5	42.2	54.2	41.8	45.9
Central	22.3	22.8	18.2	20.8	21.4
South	28.3	31.4	26.1	36.2	31.6
East	2.35	2.94	1.5	1.1	1.1
Offshore	0.66	0.66	0	0.10	0.11
Level of health service					
Primary	26.7	28.3	26.5	32.2	30.5
Secondary	53.0	47.1	50.6	49.5	51.5
Tertiary	20.3	24.6	22.9	18.3	18.0
Insulin user	0.0	12.3	4.57	3.13	5.78
CCI, mean ± SD			1.4 ± 1.8	1.6 ± 2.1	1.6 ± 2.2

^a^In total, there were 9,810 patients with DM who were free of affective disorder (AD) and were on oral anti-diabetic agents (OAA), and who could be matched for age, gender, locality, health service, Charlson Comorbidity Index and DM diagnosis date, with those who were free of AD and who were not on OAA. Where necessary, a patient in the comparison group was used more than once. Thus, there were 9,810 matched pairs. All values are percentages unless indicated.

^b^Patients diagnosed with DM and treated with metformin,(Met) who had never used insulin during the period 1996 to 2007.

^c^Patients diagnosed with DM and treated with sulfonylureas (SUs) who had never used insulin during the period 1996 to 2007.

^d^Patients diagnosed with DM and treated with both Met and SUs, who had never used insulin during the period 1996 to 2007.

**Table 2:** Total and gender-specific affective disorder (AD)^a^incidence density by diabetes mellitus (DM) status.^b^

	Total		Men		Women	
	
Descriptor	Cases/total study group, n	ID (95% CI)^c^	Cases/total study group, n	ID (95% CI)^c^	Cases/total study group, n	ID (95% CI)^c^
DM without OAAs	42/5619	74.7 (52.2 to 97.3)	11/2599	42.3 (17.4 to 67.3)	31/3019	102 (66.7 to 138)
Met only	43/5627	76.4 (53.7 to 99.2)	18/2637	68.2 (36.8 to 99.7)	25/2989	83.6 (51.0 to 116)

DM without OAAs	140/14503	96.5 (80.6 to 112)	52/7101	73.2 (53.4 to 93.1)	88/7402	118 (94.2 to 143)
SUs only	145/13370	108 (90.9 to 126)	59/6581	89.6 (66.9 to 112)	86/6788	126 (100 to 153)

DM without OAAs	282/30957	91.1 (80.5 to 101)^d^	100/14064	71.1 (57.2 to 85.0)^e^	182/16893	107 (92.2 to 123)^f^
SUs + Met	127/32236	39.4 (32.6 to 46.2)^d^	45/14791	30.4 (21.5 to 39.3)^e^	82/17444	47.0 (36.9 to 57.2)^f^

^a^AD diagnoses include ICD-9-CM (296) and the equivalent Taiwanese A-code (A212).

^a^Met, metformin; OAAs: oral anti-diabetic agents; SUs, sulfonylureas.

^c^ID (95% CI): incidence density per 10,000 person-years (95% confidence interval).

^d-f^The same superscript vertically indicates that there is a significant difference between the two groups.

The HRs for sulfonylurea and/or metformin therapy were compared with the subjects with T2DM but not on OAAs as the referent group (Table 3). Sulfonylurea or metformin alone had no significant effect on AD risk.; however, the combined treatment resulted in an evident reduction in AD risk of 60% (HR = 0.40; 95% CI 0.32 to 0.50). The findings were similar (HR = 0.42; 95% CI 0.33 to 0.53) after adjustment for monthly income and insulin usage. To eliminate any effect of insulin in combination with OAAs, a sub-analysis excluded all insulin users; the findings remained unchanged (data not shown). The order of introduction of the drugs or whether they were introduced together or separately, did not alter the significance of this finding either. Similar results were evident with both unipolar and bipolar AD, although more so with bipolar AD.

**Table 3:** Hazard ratios (HRs) and 95% confidence intervals (CIs) for affective disorders (ADs) by date of diabetes diagnosis and diabetes mellitus (DM) treatment.^a,b^

	ADs^c^			Unipolar^d^		Bipolar^e^	
	
Descriptor	Cases/total study group, n	HR (95% CI)	HR (95% CI)^f^	Cases/total study group, n	HR (95% CI)^f^	Cases/total study group, n	HR (95% CI)^f^
							
DM without OAA	42/1509	Ref.	Ref.	25/1509	Ref.	17/1509	Ref.
Metformin only	43/1509	0.92	1.00	29/1509	1.28	14/1509	0.61
		(0.59 to 1.45)	(0.61 to 1.64)		(0.68 to 2.40)		(0.22 to 1.65)

DM without OAA	140/2904	Ref.	Ref.	96/2904	Ref.	44/20904	Ref.
Sulfonylureas only	145/2904	1.08	1.13	97/2904	1.14	48/2904	1.10
		(0.84 to 1.38)	(0.87 to 1.47)		(0.83 to 1.56)		(0.68 to 1.78)

DM without OAA	282/5397	Ref.	Ref.	189/5397	Ref.	93/5397	Ref.
Sulfonylureas+ Metformin	127/5397	0.40	0.42	83/5397	0.44	44/5397	0.38
		(0.32 to 0.50)^i^	(0.33 to 0.53)^i^		(0.33 to 0.60)^i^		(0.25 to 0.58)^i^

DM without OAA	157/2843	Ref.	Ref.	109/2843	Ref.	48/2843	Ref.
SU+Met (SU first)	60/2843	0.32	0.33	36/2843	0.32	24/2843	0.37
		(0.23 to 0.44)^i^	(0.23 to 0.47)^i^		(0.21 to 0.50)^i^		(0.20 to 0.68)^i^

DM without OAA	33/968	Ref.	Ref.	18/968	Ref.	15/968	Ref.
SU+Met (Met first)	19/968	0.53	0.45	10/968	0.45	9/968	0.50
		(0.29 to 0.98)^g^	(0.23 to 0.89)^g^		(0.17 to 1.17)		(0.18 to 1.36)

DM without OAA	92/1586	Ref.	Ref.	62/1586	Ref.	30/1586	Ref.
SU+Met (the same time)	48/1586	0.50	0.54	37/1586	0.65	11/1586	0.32
		(0.35 to 0.72)^i^	(0.36 to 0.82)^i^		(0.40 to 1.04)		(0.14 to 0.75)^h^

^a^Subjects were matched by date of diagnosis (within the same calendar year), age group (50 to 54, 55 to 59, 60 to 64, 65 to 69, ≥70 uears), locality, level of care, and Carlson Comorbidity Index.

^b^OAAs: oral anti-diabetic agents; SU: sulfonylureas; Met: metformin; Ref, reference

^c^International Classification of Diseases(ICD) code 296.

^d^ICD: 296.2, 296.3,

^e^ICD: 296.0, 296.1, 296.4, 296.5, 296.6, 296.7, 296.8, 296.9

^f^Adjusted for monthly income (0, 1-15,000, 15,000-21000, >21000 NTD) and insulin usage (yes, no).

^g-i^Significance: ^g^*P*<0·05, ^h^*P*<0·01, ^i^*P*<0·001.

**Figure 5:**
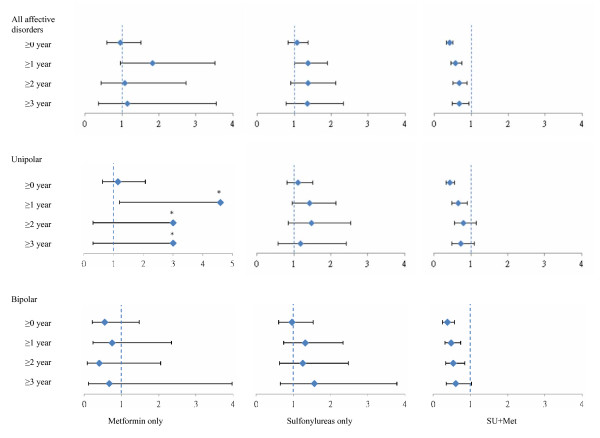
**Risk of affective disorder (AD) with sequential exclusion of its incidence for up to 3 years**. Hazard ratios (HRs) for incident ADs by date of diabetes diagnosis and type of diabetes treatment, with sequential exclusion of its incidence for up to 3 years. HRs with 95% confidence intervals (CIs) are shown. In the models used, subjects were matched by date of diagnosis (within the same calendar year), age group (50 to 54, 55 to 59, 60 to 64, 65 to 69, and ≥70 years), locality, level of care, and comorbidity index, and were adjusted for monthly income (0, 1 to 15,000, 15,000 to 21000, >21000 New Taiwan Dollars (NTD)). The upper CIs are, respectively, 17.6, 28.8, and 28.8, but bar lengths have been omitted in the figure because of scale.

Although subjects were matched for CCI to minimize any effect on incident AD of underlying or latent disease at the time of diabetes treatment initiation, the incidences for the first 3 years were progressively excluded (Figure 5). The HRs for combined sulfonylurea and metformin remained significant into the third year for all incident AD, at 0.67 (0.48 to 0.93). The findings are similar for both bipolar and unipolar AD, but there was a trend toward a greater reduction in bipolar AD.

## Discussion

### Affective disorder and diabetes in Taiwan

In this study, we found that for Taiwanese subjects with T2DM aged 50 years or over, having no oral anti-hyperglycemic therapy is a risk factor for AD, with an overall increase in risk of 2.62-fold during the period 1996 to 2007. One reason our findings are not negative [11], unlike those of other studies [12-15], may be that they were not masked by therapeutic interventions for diabetes that we have previously found to reduce the risks of dementia [23] and Parkinson's disease [24]. Another is that in Taiwan, as elsewhere, the burden of diabetes in an ageing population is increasing [17], and thus the phenomenon is more recognizable. However, in some community-based ageing populations such as that of Australia, older people do not necessarily show an increased prevalence of depression [32]. It is possible that diabetes might play a greater role in the development of depression or AD in communities in which its prevalence increases to a greater extent in later life. Gender did not seem to influence the status of diabetes as a risk factor for AD, whether unipolar or bipolar. This does not diminish the importance of gender differences in risk for depression or AD that have been recognized for other factors [32], or of the pathogenetic and clinical differences between the major types of AD, but it does emphasize the possible commonalities through the diabetes linkage and raises questions about which of these might be relevant in prevention and management.

There has been no study to date of T2DM and AD on East Asian populations, where marked increases in T2DM prevalence have occurred in recent years, and where almost half of the world's population is exposed to diabetes, and consequently to a potential threat to mental health. Our study makes a contribution to the relevance of diabetes as a risk factor for AD in East Asian populations, as Taiwan has a dominantly Chinese ethnic population and is therefore an example of a large under-studied group.

### Preventive or therapeutic benefit of oral anti-hyperglycemic agents for affective disorder

The available studies have not considered diabetes pharmacotherapy in relation to the effect of diabetes on the risk for depression or AD, which may have led to lower estimates of the possible reduction in risk of AD by diabetes therapy. In the meta-analysis on Europeans and North Americans carried out by Nouwen *et al. *[12], the increased risk for depression was 24% and in the study by Nefs *et al. *[14], it was 14%; given our findings, these were probably underestimates. We found the increased risk for incident AD in subjects with T2DM untreated by OAA to be 2.6-fold (both unipolar and bipolar AD HRs were similar, at 2.81 and 2.31, respectively), which suggests that the available studies have grossly underestimated the potential effect of diabetes in this area of mental health.

The combination of sulfonylurea and metformin significantly reduced both incidence and relative risk for AD in T2DM, irrespective of gender; there was no such change evident for sulfonylurea or metformin alone (Table 2, Table 3). It also did not matter in which order sulfonylurea or metformin were started. For any particular intervention, patients were extensively matched with their reference non-OAA subject, using counterparts with the same date of diabetes diagnosis, within the same calendar year. This reduced the possibility that a change in accepted diabetes management might have accounted for the observed effects, whether in men or women. Bias, especially from time, was minimized, and thus made this cohort study more similar to a clinical trial [29,30]. Differences in referent subjects between treatment categories were addressed by matching for matters such as accessibility to care (by locality), level of care (by primary to tertiary or medical center) and associated health status (CCI) (Table 3).

Despite adjustment for co-morbidities and the exclusion of incident AD for up to 3 years after introduction of OAA therapy, the combination of sulfonylurea and metformin was still associated with HRs of less than 1.00 compared with no OAA therapy. This applied to both unipolar and, to a greater extent, bipolar AD. Thus, although differences between these two forms of AD are well documented and with agreed therapies, our findings raise the possibility that there are some common underlying pathogenetic factors, perhaps related to neurodegeneration.

The findings for the sulfonylurea/metformin combination rather than either alone would be consistent with synergistic or complimentary mechanisms of action of the two agents. Because advanced glycation end-products are found in the central nervous system (CNS), specifically in microglia [33], these two OAAs together might act via a more pronounced anti-hyperglycemic effect. Such an effect might be combined with specific AD-reduction actions of the drugs. As mentioned above, metformin might operate through mechanisms that are protective against neurodegeneration, if this is a shared pathogenesis with AD. When diabetes affects the CNS, AD might share underlying mechanisms with neurodegenerative diseases such as dementia and Parkinsonism [23,24]. We previously reported that T2DM also increases the risk of dementia by 2.6-fold, and that the combination of sulfonylureas and metformin was able to decrease the risk of dementia in diabetes within 12-years [23], with similar findings in PD [24]. These collective findings strengthen the case for a linkage between brain diabetes, neurodegenerative disease, and AD, a conjunction already in evidence from various pathophysiological studies [33,34]. Chen *et al*.. found that metformin might increase the biogenesis of amyloid peptides in Alzheimer's disease, which could be relevant if there is a shared pathogenesis with depression, so our population-wide study supports this idea [35].

The definition of OAA usage in our models was a prescription for at least 3 months. Minimal doses for metformin were equivalent to about 500 mg per day and, for sulfonylureas, one standard tablet or capsule per day. These therapeutic regimens are feasible for most people with T2DM, and may largely remove the risk of AD posed by diabetes within 12 years of starting treatment. Owing to the limited number of subjects with T2DM on insulin therapy in this study, we did not treat them separately. Controlling for or exclusion of insulin usage in the models made no difference to our findings about OAAs and AD.

### Issues in the diagnosis of affective disorder in diabetes

The Taiwanese NHI claim data rely on the medical service utilization of patients and on medical diagnoses. Underdiagnosis of AD and DM is likely if the reach of a healthcare system is limited, but NHI covers more than 98% of the population in Taiwan and, with the use of our matching protocol for accessibility (that is, region, level of service, and income), underdiagnosis should have been minimized. For social reasons, AD may be under-reported, but this is less likely with diabetes because of frequent and more intensive contact with the health system. Nevertheless, clinicians may have a tendency to simplify the diagnostic list when patients have multisystem diseases such as diabetes, in favor of the 'less pressing' diagnoses, which might therefore mean less recording of AD.

We have no information about the diagnosis of either diabetes or AD before 1996, when the NHI started, but it is unlikely that the presence of either of these would not have been recorded by a medical practitioner at some subsequent consultation. However, because the diagnosis of bipolar AD depends on at least two visits, such conditions may have been under-diagnosed as a consequence. Thus, there are several factors which may affect AD diagnosis rates, and thus their net effect on the differentials with regard to antecedent diabetes remains unclear.

A valuable insight into the validity of these diagnostic rates comes from the relevance of diagnostic methods, whether questionnaire or psychiatric diagnosis [12]. Differences in estimates have been found to be dependent on year of publication of the report, but also to be probably increasing with time. Thus, there are questions about how contemporary our work, using 1996 datasets, might be regarded, because, as an NHI system requirement, we used ICD-9 for the diagnosis of AD rather then ICD-10 or DSM-IV. The ICD-9 codes used in the present study were 296.0 to 296.9 and its Taiwanese coding counterpart was A212 (before year 2000); nevertheless, these do cover ICD-10 codes F30 to F33 and F38 to F39, and thus, our study should be replicable.

We recognize that using the DSM-IV would have been a more useful approach to the diagnosis of depression and, more particularly, mood or AD, because it includes clinical symptoms and other axes that would have provided more analytical scope. However, it is reassuring that there can be some convergence of the risk assessment for diabetes on depression or AD, even using different methods [12]. At the same time, our large and representative population-based study has allowed a more detailed understanding of the association of diabetes with different mood disorders than would otherwise have been possible.

### Limitations

In our study of AD, we considered both its unipolar and bipolar forms, but not dysthymic disorders, thus our findings are not applicable to all forms of depression. With regard to diabetes as a risk factor for AD, there seems to be no gender difference. Nor did we find any apparent difference between unipolar and bipolar AD, although sample size may have been limiting. Because incident AD was progressively excluded in the models, sample size diminished. Therefore, we may not have been able to discern more substantial and longer-term differences between unipolar and bipolar AD insofar as OAA effects are concerned.

T2DM and depression or AD seem to be at least bidirectional, if not mutually reinforcing [10,36]. This process may begin at an early stage in the evolution of diabetes from pre-diabetic states and in the early stages of depressive illness. We did not study pre-diabetes subjects, but we did track individuals through what will have been that phase, from being diabetes-free in the 1996 to 2007 cohort. From other evidence [10,36], we cannot fully exclude effects on depression-induced diabetes, which may have, in turn, increased AD incidence.

The study cohort is an administrative sample for which measures of diabetes severity are not available. Therefore, we do not have dose-response data for the studied OAAs and glycemic control. However, we can say that this particular OAA combination changes the risk for AD in T2DM, what the minimal dosages are likely to be for protection against AD, and how long it might take for these effects to be evident, as these informed the method we used to establish exposures. Matching for CCI, which partly represents diabetes-related complications and its severity, make it more likely that we observed a genuine OAA effect rather than one simply of glycemic control, although the latter is still possible.

Although ours is not an intervention study, we found at least one OAA combination which could enhance risk reduction for AD. This is unlikely to be a surrogate for other potential determinants of risk like body mass index. Moreover, were this combination of sulfonylurea and metformin to operate to reduce AD risk through a possible surrogate like body mass index, it might well be that these OAA act in directions opposite from each other, given their known opposite effects on body mass index. Unfortunately, these covariates are unavailable.

## Implications

It is difficult to say how early or late in the course of diabetes it may be possible for OAAs to reduce the risk of AD, but if the association is causal and has an underlying pathogenesis shared with neurodegeneration, then the responsive period is likely to be earlier rather than later. Therefore, the early recognition of those at risk for diabetes may allow reduction in the burden of depression. It is now recommended that metformin should form part of the therapeutic management of T2DM as early in its course as possible because of the superior cardiovascular and all-cause mortality outcomes with this drug compared with other OAAs [37]. We consider that a broader view of diabetes comorbidity and its management will stimulate interest in changing diabetes therapeutics, with the prospects of a significant reduction in the burden of disease seen with the tandem of diabetes and AD.

## Conclusions

In a large cohort representative of the Taiwanese population, followed for up to 12 years, T2DM increased the risk of AD 2.6-fold. However, the HR for AD for patients taking combined sulfonylurea and metformin remained significantly decreased into the third year for all incident AD at 0.67 (0.48 to 0.93), and this was evident for both bipolar and unipolar AD. As the global burden of diabetes increases, these findings may be relevant to the reduction of its mental-health complications.

## Abbreviations

AD: affective disorders; CCI: Charlson Comorbidity Index; CI: confidence interval; CNS: central nervous system; HR: hazard ratio; NHI: National Health Insurance; OAA: oral anti-hyperglycemic agent; T2DM: type 2 diabetes.

## Competing interests

The authors declare that they have no competing interests.

## Authors' contributions

MLW, MSL, CCH, and SYC designed the study; MLW, MSL and CCH managed the study; MLW, CCH, MSL, and HNT analyzed and interpreted the data; MLW wrote the paper; MLW, MSL, SYC, CCH, HNT, SHY and HYC read, revised and approved the final manuscript

## Pre-publication history

The pre-publication history for this paper can be accessed here:

http://www.biomedcentral.com/1741-7015/10/150/prepub
